# Genetic Analysis Algorithm for the Study of Patients with Multiple Congenital Anomalies and Isolated Congenital Heart Disease †

**DOI:** 10.3390/genes13071172

**Published:** 2022-06-29

**Authors:** Marisol Delea, Lucia S. Massara, Lucia D. Espeche, María Paz Bidondo, Pablo Barbero, Jaen Oliveri, Paloma Brun, Mónica Fabro, Micaela Galain, Cecilia S. Fernández, Melisa Taboas, Carlos D. Bruque, Jorge E. Kolomenski, Agustín Izquierdo, Ariel Berenstein, Viviana Cosentino, Celeste Martinoli, Mariana Vilas, Mónica Rittler, Rodrigo Mendez, Lilian Furforo, Rosa Liascovich, Boris Groisman, Sandra Rozental, Liliana Dain, on behalf of the PID ACM-CC Group

**Affiliations:** 1 Centro Nacional de Genética Médica “Dr. Eduardo Castilla”- ANLIS “Dr. Carlos G. Malbrán”, Avda. Las Heras 2670, Buenos Aires 1425, Argentina; marisoldelea@gmail.com (M.D.); luciadespeche@gmail.com (L.D.E.); mariapazbidondo@gmail.com (M.P.B.); pablobarbero63@hotmail.com (P.B.); mtaboas@anlis.gov.ar (M.T.); bruquecarlos@gmail.com (C.D.B.); rodrigomendezh@gmail.com (R.M.); rosaliascovich@hotmail.com (R.L.); bgroisman@gmail.com (B.G.); sandrarozental@yahoo.com.ar (S.R.); 2 Hospital de Alta Complejidad en Red El Cruce—SAMIC. Av. Calchaquí 5401, Florencio Varela 1888, Argentina; luciasolemassara@gmail.com (L.S.M.); jaenoliveri@gmail.com (J.O.); brunpaloma@gmail.com (P.B.); 3 Unidad Académica de Histologia, Embriologia, Biologia Celular y Genética, Facultad de Medicina UBA, Paraguay 2155, Buenos Aires 1121, Argentina; 4 Novagen, Viamonte 1430, Buenos Aires 1055, Argentina; mfabbro@novagen.com.ar (M.F.); mgalain@novagen.com.ar (M.G.); cecilia.fernandez@novagen.com.ar (C.S.F.); 5 Departamento de Fisiología, Biología Molecular y Celular, Instituto de Biociencias, Biotecnología y Biología Traslacional (iB3), Facultad de Ciencias Exactas y Naturales-UBA, Intendente Güiraldes 2160, Buenos Aires 1428, Argentina; ekolomenski@gmail.com; 6 Centro de Investigaciones Endocrinológicas “Dr. César Bergadá”. Gallo 1330, Buenos Aires 1425, Argentina; aguizquierdo@gmail.com; 7 Instituto Multidisciplinario de Investigaciones en Patologías Pediátricas, Gallo 1330, Buenos Aires 1425, Argentina; arieljberenstein@gmail.com; 8 Hospital Interzonal General de Agudos Luisa Cravenna de Gandulfo, Balcarce 351, Lomas de Zamora 1832, Argentina; viviconsentino@hotmail.com; 9 Hospital Sor Maria Ludovica, Calle 14 1631, La Plata 1904, Argentina; celestemartinoli@yahoo.com; 10 Hospital Materno Infantil Ramón Sardá, Esteban de Luca 2151, Buenos Aires 1246, Argentina; marianavilas@hotmail.com (M.V.); rittlerm@gmail.com (M.R.); lilianfurforo@gmail.com (L.F.)

**Keywords:** multiple congenital anomalies, congenital heart disease, chromosomal abnormalities, array-CGH, next-generation sequencing

## Abstract

Congenital anomalies (CA) affect 3–5% of newborns, representing the second-leading cause of infant mortality in Argentina. Multiple congenital anomalies (MCA) have a prevalence of 2.26/1000 births in newborns, while congenital heart diseases (CHD) are the most frequent CA with a prevalence of 4.06/1000 births. The aim of this study was to identify the genetic causes in Argentinian patients with MCA and isolated CHD. We recruited 366 patients (172 with MCA and 194 with isolated CHD) born between June 2015 and August 2019 at public hospitals. DNA from peripheral blood was obtained from all patients, while karyotyping was performed in patients with MCA. Samples from patients presenting conotruncal CHD or DiGeorge phenotype (n = 137) were studied using MLPA. Ninety-three samples were studied by array-CGH and 18 by targeted or exome next-generation sequencing (NGS). A total of 240 patients were successfully studied using at least one technique. Cytogenetic abnormalities were observed in 13 patients, while 18 had clinically relevant imbalances detected by array-CGH. After MLPA, 26 patients presented 22q11 deletions or duplications and one presented a *TBX1* gene deletion. Following NGS analysis, 12 patients presented pathogenic or likely pathogenic genetic variants, five of them, found in *KAT6B*, *SHH*, *MYH11*, *MYH7* and *EP300* genes, are novel. Using an algorithm that combines molecular techniques with clinical and genetic assessment, we determined the genetic contribution in 27.5% of the analyzed patients.

## 1. Introduction

Congenital anomalies (CA) are prenatal clinically significant birth defects resulting from morphological disturbances in the process of human development affecting infant morbidity and mortality, regardless of their pathogenesis, etiology, and time of diagnosis [1]. CA affect 3–5% of newborns and may be classified as major or minor. Major CA are those that have significant medical, social, or cosmetic consequences and typically require medical intervention, such as spina bifida or cleft palate. Minor CA are more prevalent in the population and represent structural changes that do not pose significant health problems in the neonatal period and tend to have limited social or cosmetic consequences for the affected individuals [1]. Examples include single palmar crease and clinodactyly. CA are primarily isolated but nearly 20–30% of infants with CA have multiple congenital anomalies (MCA) involving major anomalies in different organs and systems [2].

Among CA, congenital heart defects (CHD) are the most common type of birth defect with a worldwide prevalence of approximately 9 per 1000 births, representing the leading cause of mortality in the first year of life [3,4]. In addition, around 1 in 200 infants has MCA [5]. These patients have serious medical, as well as familial and social implications, including early and high lethality [6,7].

In Argentina, CA represents the second-leading cause of infant mortality, following perinatal conditions. MCA prevalence at birth is 2.26 per 1000 births, whereas CHD are the most frequent CA, with a prevalence at birth of 4.06 per 1000 births [8].

The etiology of these defects is widely recognized as heterogeneous, with a contribution of genetic and environmental factors. Around 5% to 10% of CA are due to environmental and maternal causes, such as teratogenic agents (i.e., alcohol), malnutrition, drug or toxin exposure, and maternal infection and disease, whereas around 40% are known to have a direct genetic cause, either chromosomal, multifactorial, or single-gene defects [9,10,11]. Numerical and structural chromosomal abnormalities account for approximately 15% of patients with major CA [12]. Microdeletions and microduplications have been described in 10–17% of MCA patients [13,14]. Among them, the 22q11 deletion syndrome (22q11DS), also known as Velocardiofacial (VCF)/DiGeorge syndrome, represents the most common microdeletion syndrome in humans. While conotruncal CHD is one of the most common phenotypic manifestations in 22q11DS, this deletion was also found in a significant number of patients with isolated conotruncal CHD [15,16,17]. Finally, single-gene defects account for a number of well-recognized syndromes (https://www.omim.org/statistics/geneMap, last accessed on 10 December 2021) [18] as well as 3–5% of patients with CHD [19]. Nevertheless, in a significant number of patients, the etiology still remains unknown [20,21].

Although largely studied in several populations, the genetic contribution to CA in Latin America is less documented [22,23,24,25,26,27,28]. Considering their severity and birth prevalence, the aim of this study was to identify the genetic causes in Argentinian patients with MCA and isolated CHD.

## 2. Materials and Methods

### 2.1. Patients

A total of 366 patients (172 MCA and 194 isolated CHD) were recruited between June 2015 and August 2019 from different public institutions: 13 hospitals participating in the National Network of Congenital Anomalies of Argentina (RENAC) from Buenos Aires city and Buenos Aires Province, and the genetic services of Sor María Ludovica Hospital and El Cruce Hospital, both from Buenos Aires Province.

The patients were evaluated by a neonatologist, a cardiologist, and/or a clinical geneticist. A complete physical examination was performed, and a detailed individual and familial history was retrieved. Case definition of MCA included: two or more major unrelated morphological CA, externally or internally located, detected by physical examination, complementary tests, or surgery. Case definition of isolated CHD included: one or more heart defects detected by physical examination and confirmed by echocardiogram, without the presence of other extracardiac anomalies. For the present study, cases with Down Syndrome phenotype, newborns <37 weeks of gestation with ductus, and newborns with foramen ovale independent of the gestational age, were excluded. The female/male ratio was: 1.03 (86/83, 3 with ambiguous genitalia) for patients with MCA and 1.02 (98/96) for isolated CHD.

### 2.2. Algorithm Applied

**Figure 1:**
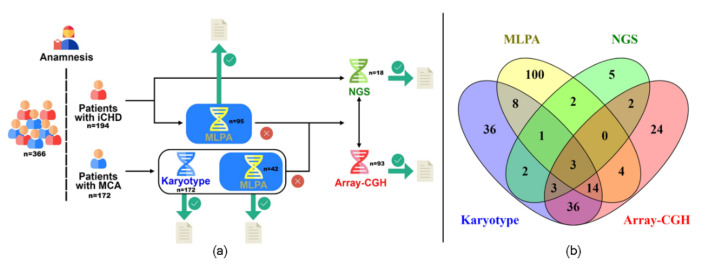
Different approaches applied to patient analysis. (**a**): Schematic representation of the algorithm applied for patients’ analysis. (**b**): Venn diagram showing the number of successfully analyzed samples through the algorithm described in (**a**). iCHD: Isolated congenital heart defect; MCA: Multiple congenital anomalies; MLPA: Multiplex-dependent ligation probe amplification; Array-CGH: array comparative genomic hybridization; NGS: Next-generation sequencing.

We designed an algorithm to be applied sequentially based mainly on the possibility of achieving the highest chance of finding the genetic cause in the analyzed samples. Figure 1a shows the algorithm applied. DNA from peripheral blood was obtained from all patients, whereas karyotyping was performed for those presenting with MCA (n = 172). Samples from patients affected with conotruncal CHD (n = 105, 26 with MCA, 79 with isolated CHD) were analyzed by Multiplex-dependent Ligation Probe Amplification (MLPA). MLPA analysis was also applied for patients with clinical characteristics compatible with 22q11DS regardless of the presence of conotruncal CHD (n = 32, 16 with MCA, 16 with isolated CHD). Therefore, a total of 137 samples were studied by MLPA (42 patients with MCA and 95 patients with isolated CHD). Ninety-three samples from patients presenting MCA were selected for chromosomal microarray analysis (CMA). Eligibility criteria were based on: (1) balanced karyotype, (2) failed or normal karyotype with a high number of congenital anomalies in the patient, and (3) cytogenetic anomaly which required a more precise delineation of chromosomal breakpoints.

Eighteen patients were selected for targeted or exome next-generation sequencing (NGS), based on: (1) suspected monogenic syndrome, (2) familiar history, and (3) inconclusive phenotype/genotype correlation after CMA.

### 2.3. Cytogenetic Analysis

Cytogenetic analysis was performed in peripheral blood lymphocytes by trypsin-Wright (GTW) banding technique according to standard procedures. The International System for Human Cytogenomic Nomenclature 2020 (ISCN) was used for nomenclature reference [29]. In selected samples, Fluorescent in Situ Hybridization (FISH) was performed to further confirm the cytogenetic findings. When necessary, and if available, karyotypes were also performed on blood samples from parents.

### 2.4. Multiplex Ligation-Dependent Probe Amplification Analysis (MLPA)

MLPA analysis was performed using the SALSA P250-B1 MLPA kit (MRC-Holland, Amsterdam, The Netherlands) as previously described [28].

### 2.5. Chromosomal Microarray Analysis (CMA)

Among the 93 patients selected for array-CGH (array comparative genomic hybridization) analysis, 89 samples were studied with the ISCA v2 8×60K platform and 4 with ISCA 4×180K (Agilent, Santa Clara, CA, USA) platform, as previously described [30,31]. For the categorization of the CNVs, the American College of Medical Genetic and Genomics (ACMG) and ClinGen technical standards for interpretation and reporting of constitutional CNVs [32] were followed. Imbalances were grouped into five categories: (1) pathogenic, (2) likely pathogenic, (3) variant of uncertain significance (VUS), (4) likely benign, and (5) benign. In some cases, familial samples were also analyzed for a full interpretation of the proband’s microarray result. Genomic imbalances were annotated based on the GRCh37/hg19 Genome Build (February 2009). The International System for Human Cytogenomic Nomenclature 2020 (ISCN) was used for nomenclature reference [29].

### 2.6. Next-Generation Sequencing (NGS) Analysis

Approximately 1 ug of DNA from each of the 18 selected patients was analyzed by targeted NGS or whole-exome sequencing (WES). Targeted NGS was performed in 6 CHD patients (4 with isolated CHD, 2 with MCA) with the aid of the TruSight^®^ Cardio Sequencing kit (Illumina, San Diego, CA, USA) [33]. In 12 patients with MCA, WES was performed using the Agilent SureSelect Human All Exon V6 and V7 kits (Agilent Technologies, Santa Clara, CA, USA) followed by an in-silico selection of candidate genes for variant analysis. Phenotype-driven gene lists of interest were developed in-house for each case based on Human Phenotype Ontology (https://hpo.jax.org/app/ last accessed on 10 December 2021), OMIM (https://www.omim.org/ last accessed on 10 December 2021), ClinVar (https://www.ncbi.nlm.nih.gov/clinvar/ last accessed on 10 December 2021), ORPHANET (https://www.orpha.net/ last accessed on 10 December 2021) and PanelApp (https://panelapp.genomicsengland.co.uk/panels/ last accessed on 10 December 2021). In addition, a literature search was performed in PubMed (https://www.ncbi.nlm.nih.gov/pubmed/ last accessed on 10 December 2021) to find known genes that most frequently presented variants in patients with the phenotype under study. Keywords of the suspected syndromes, HPO terms and/or embryonic structures involved were used for searching. In addition, a search was conducted for genes involved in the development of affected structures irrespective of the presence of pathogenic variants in patients.

Variant prioritization was based on inheritance patterns, variant type, population frequencies, affected gene, functional impact prediction, sequence conservation, pathogenicity predictors, and information on clinical databases. Variants were interpreted using American College of Medical Genetics and Genomics (ACMG) guidelines [34]. The general assertion criteria for variant classification are publicly available on the GeneDxClinVar submission page (http://www.ncbi.nlm.nih.gov/clinvar/submitters/26957 last accessed on 10 December 2021/). All identified sequence changes of interest were confirmed by Sanger sequencing and, when available, segregation within the family was also determined. Genetic variants were noted following the recommendations of the Human Genome Variation Society (HGVS) [35].

All clinically relevant CNVs and genetic variants were uploaded to the ClinVar database (accession # SCV002074131, SCV002098068–SCV002098099, SCV002098932).

## 3. Results

We recruited a total of 366 patients presenting MCA or isolated CHD. The putative genetic etiology was assessed in 240 patients (143 MCA and 97 isolated CHD), successfully analyzed at least by one technique (Figure 1b), while the remaining could not be studied due to different factors such as unavailable or insufficient quality of samples. Altogether, the algorithm applied allowed us to determine the genetic contribution in 66 of these 240 patients (27.5%), 43 (30%) with MCA and 23 (23.7%) with isolated CHD.

### 3.1. Cytogenetic Analyses

Of the 172 MCA patients included, 103 were successfully karyotyped and 13 (12.6%) presented a cytogenetic abnormality (Table 1). In the remaining 69 samples, karyotyping was not performed due to culture failure or insufficient chromosome quality for analysis.

### 3.2. Multiplex Ligation-Dependent Probe Amplification Analysis (MLPA)

A total of 132 out of the 137 samples included with conotruncal CHD or clinical characteristics compatible with 22q11DS were successfully analyzed by MLPA. Table 2 summarizes the obtained results. As shown, 27 (20.5%) patients presented at least one imbalance: 24/102 (23%) with conotruncal CHD and 3/30 (10%) compatible with 22q11DS, but without conotruncal CHD. Although none of these three patients presented conotruncal CHD, each one exhibited an isolated CHD: pulmonary branch stenosis, vascular ring, and ventricular septal defect (VSD) with dilated cardiomyopathy, which were already described as associated with 22q11DS as well [36]. Of the total of 27 patients, 24 had 22q11 deletion (21 comprising the typical 3 Mb deletion and 3, the shorter 1.5 Mb deletion), two had a 1.5 Mb duplication, and one had a partial *TBX1* deletion.

### 3.3. Chromosomal Microarray Analysis (CMA)

A total of 86 DNA samples from patients with MCA out of 93 selected were able to be assessed by array-CGH. We found 19 clinically relevant CNVs (pathogenic or likely pathogenic) in 18 patients (21%) (see Supplementary Figure S1 for an ideogram of the chromosomal localization of these CNVs). Table 3 shows the clinically relevant CNVs found, and the details of patients’ phenotypes are summarized in Supplementary Table S1. In addition, we found 6 CNVs classified as VUS in 6 patients (Supplementary Table S2).

Patient IDs 48 and 368 were included for CMA analysis to better define the cytogenetic anomaly observed. CMA analysis revealed no imbalances involving the regions of the t(1;2)(q25;q21) in patient ID 48. Instead, a deletion in 8q21.11q21.3 was found. In patient 368, CMA showed the presence of a tetrasomy of the short arm of chromosome 8.

Patient ID 100 presented Megacystis-Microcolon-Intestinal Hypoperistalsis Syndrome (MMIHS), an autosomal recessive disorder, and was formerly analyzed by NGS (see below). The genetic variant found by massive sequencing, a 5 bp in the *MYH11* gene, was in an apparently homozygous state and inherited from her heterozygous mother. To further exclude a putative hemizygous state encompassing the *MYH11* gene, we analyzed the proband and the parents by CMA. Our results showed the presence of a 0.6 kb deletion in 16p13.11 encompassing *MYH11* on the paternal allele.

Patient ID 149 has a complex phenotype presenting MCA and familial intestinal polyposis. CMA revealed a 0.02 Mb deletion encompassing the *APC* gene. Considering that the deletion in *APC* is consistent with the intestinal polyposis but not with the MCA, we also performed NGS analysis (see below) for this patient.

### 3.4. Next-Generation Sequencing (NGS)

Following NGS analysis, 12 out of 18 (67%) patients presented pathogenic or likely pathogenic genetic variants. Five of the variants, found in the *KAT6B*, *SHH*, *MYH11*, *EP300,* and *MYH7* genes, are novel. Table 4 summarizes the clinically relevant genetic variants found. Supplementary Figure S2 shows Sanger sequencing and segregation results (when available) from all the clinically relevant genetic variants. Details on patients’ phenotypes are summarized in Supplementary Table S3.

**Table 3:** Details on the clinically relevant CNVs found in patients presenting MCA.

Patient ID	Karyotype	Imbalance	Size (Mb)	Classification	OMIM #	ORPHA #	Supporting Evidence
2	46,XX	arr[GRCh37] 2q24.2q31.1(160347642_174075851)x1	13.73	Pathogenic	-	1617	[37,38]
41	46,XY	arr[GRCh37] 7q11.23(72766313_74042787)x3	1.27	Pathogenic	609757	261102	[39]
48	46,XY,t(1;2)(q25;q21) ^1^	arr[GRCh37] 8q21.11q21.3(75904944_87097083)x1	11.19	Pathogenic	614230	284160	[40]
65	Failed	arr[GRCh37] 7q36.1q36.3(149062717_159124131)x1	10.06	Pathogenic	-		[41,42,43]
68	Failed	arr[GRCh37] 2q14.2q14.3(120628484_127658188)x1	7	Pathogenic			[44]
94	47,XXX [28]/47,XX,+14 [12]	arr(14)x3,(X)x3	-	Pathogenic	-	-	[31]
96	46,XX	arr[GRCh37] Xp22.33(940688_2676609)x3	1.7	Pathogenic	-	-	[45]
100 ^2^	Failed	arr[GRCh37] 16p13.11(15551302_16194578)x1pat	0.64	Pathogenic	619351	2241	[46,47]
106	46,XX	arr[GRCh37] 17q25.3(80583397_81044553)x1	0.46	Likely Pathogenic	-		[48,49]
127	46,XY	arr[GRCh37] 16p12.2(21837492_22407931)x1	0.57	Pathogenic	136570		[50]
134	Failed	arr[GRCh37] 1p36.33p36.23(834101_7930605)x1; 7q35q36.3(146927174_159128556)x3 ^1,3^	7.1; 12.2	Pathogenic	607872,-	1606	[51,52,53,54]
147	46,XY	arr[GRCh37] 15q14(33809650_40027263)x1	6.22	Pathogenic	616898	261190	[55,56]
149 ^4^	46, XY	arr[GRCh37] 5q22.2(112155123_112174165)x1pat	0.02	Pathogenic	-	261584	[57,58]
167	Failed	arr(13)x3	-	Pathogenic	-	3378	[59]
187	Failed	arr(18)x3	-	Pathogenic	-	3380	[60]
233	46,XY	arr[GRCh37] 9q22.2q31.1(93864974_106661581)x1	12	Pathogenic	109400		[61]
362	Failed	arr[GRCh37] 3p21.31(44948482_49115809)x1dn	4.1	Pathogenic	-	-	[62,63,64,65]
368	46,XY,trp(8)(p21.1p21.2)	arr[GRCh37] 8p21.3p21.2(19779604_26531980)x4	6.7	Pathogenic	-	-	[66]

^1^: Parents presented a normal karyotype. ^2^: This patient was initially studied by NGS (see Table 4). ^3^: FISH analysis: ish der(1)t(1;7)(p36;p35)(subtel1p-,subtel7q+,subtel1q+). ^4^: This patient was also studied by NGS (see Table 4). Failed: culture failure or insufficient chromosomal quality.

All the genetic variants were found in a heterozygous state, except for the 5 bp deletion c.3143-2_3145delAGTGC in *MYH11* in the proband ID 100 which was found in an apparently homozygous state, further confirmed by Sanger sequencing in the proband and in her affected sister ID 100H1. The variant was inherited from their heterozygous mother while the father presented only the wild-type sequence (Supplementary Figure S2). CMA confirmed the hemizygous state of this region on the paternal allele (see above). Both parents were healthy. The 5 bp deletion found in the probands comprises 2 bp of the canonical acceptor splicing site in the intron 25 and 3 bp of the exon 26 of the *MYH11* gene.

Patient ID 123 presented a genetic variant in *FOXL2* inherited from the father who also presented Blepharophimosis, Ptosis, and Epicanthus Inversus syndrome (BPES) as the proband.

The genetic variant in the *KAT6B* gene found in the patient from our cohort ID 188 has been described previously [81].

We identified a genetic variant in the *EP300* in patient ID149. Sanger sequencing revealed the inheritance of the variant from his father, as was the 0.02 Mb pathogenic deletion at 5q22.2 (see Table 3).

Finally, two unrelated patients presented the same genetic variant in the *MYPBC3* gene. In both probands, the variant was inherited from their mothers.

## 4. Discussion

The etiology of CA is widely recognized as heterogeneous with the contribution of genetic and environmental/maternal factors [9,11]. In this work, we applied several approaches to ascertain the putative genetic causes related to CA in a group of patients from Argentina. To the best of our knowledge, the present report would also be the first study in our country applying CMA and NGS in a cohort of patients with MCA and isolated CHD. The selection of these two groups of patients was based on their severity and birth prevalence. CHD represent the leading cause of mortality in the first year of life [3,4], while infants born with MCA have serious medical implications with early and high lethality [6,7]. In our country, patients born with MCA and CHD represent 21% and 29% of all CA, respectively, and approximately 57% of the infants with CHD elicited an isolated CHD (Consulted to The National Network of Congenital Anomalies of Argentina (RENAC) database, period 2009–2020).

We designed and implemented a sequential algorithm to offer the most suitable and appropriate approach to elucidate the putative genetic cause in the affected patients. Cytogenetic analysis was used in the present work as the first-tier genetic test for patients referred with MCA. Even though microarray analysis is largely recommended as a first-tier test for this group of patients [87], CMA is not widely available in the public health sector of Argentina, mainly due to financial limitations. Accordingly, we also applied a phenotype-first approach to select a group of patients that would benefit the most from the analysis by NGS, also considering the above-mentioned limitations of this methodology in our country. This approach consisted of a decision-making process based on the probability of presenting a pathogenic variant. This probability increases if the phenotype is compatible with a known genetic syndrome or if there is a known genetic cause implicated in pathogenesis.

Applying this sequential algorithm, which also includes MLPA analysis for patients presenting conotruncal CHD or clinical characteristics compatible with 22q11DS, we define the genetic contribution in 66 patients: 43 MCA and 23 isolated CHD.

After cytogenetic analysis, the number of patients with numerical and structural chromosomal abnormalities (13/103; 12.6%) in our study is similar to other results, showing that approximately 15% of patients with major CA have cytogenetic abnormalities [12]. However, it should be noted that approximately 40% of the samples studied by cytogenetic analysis remained unsolved due to culture failure or chromosomal quality, mainly those referred to in the neonatal period. In fact, we were able to define the genetic cause in some of these patients after CMA (Table 3), reinforcing the importance of applying CMA analysis routinely to overcome technical difficulties in cytogenetic studies. CMA was also applied to samples presenting a balanced karyotype to ascertain gain/loss of DNA material (see below), and in a sample with an apparent triplication of the short arm of chromosome 8, to better define the rearrangement. CMA showed that this patient presented a tetrasomy of the short arm of chromosome 8 (Table 3). Tetrasomy 8p is a very rare chromosomal abnormality. To our knowledge, only 15 patients have been reported, all of them presenting a mosaic isochromosome 8p detected postnatally [66]. Thus, this would be the first report of a patient presenting a non-mosaic partial tetrasomy 8p. His phenotype resembled most of the features already described, except for the presence of seizures, with only one proband reported with severe epilepsy [88].

Unfortunately, for the patient with 46,XX,t(11;17)(p10;p10) karyotype, the definitive genetic etiology remains unsolved. The DNA sample was initially not available for CMA and a new sample could not be obtained since the patient died shortly after birth. In the remaining 10 patients in which chromosomal abnormalities were found, cytogenetic findings were in accordance with their phenotypes

We found 22q11 imbalances in 23% of the patients analyzed by MLPA with conotruncal CHD, similar to our previous results [28]. Although the 22q11 imbalances were most prevalent among patients presenting MCA, 22% of the patients with isolated conotruncal CHD had a 22q11 microdeletion or duplication, similar to other reports [15,16,17,89]. For these patients, early diagnosis and interventions are key for the management of putative late complications such as immunodeficiency, hypocalcemia, developmental and speech delay, behavioral phenotypes, and psychiatric illness [90]. Most of these phenotypic characteristics may not be evident at birth or in early childhood and outline the importance of genetic screening of patients with conotruncal CHD even in the absence of other anomalies.

The advent of technologies that allowed whole-genome analysis like CMA and NGS and its inclusion into medical practice has contributed to the identification of genetic causes related to CA. Association between pathogenic CNVs in patients presenting MCA and non-syndromic CA has been largely described [7,13,91,92,93,94,95,96,97], although only a few large cohort studies have been specifically performed aiming at analyzing the whole genome by array-CGH in samples with birth defects in Latin American populations [93]. Similarly, NGS data from patients with CA from our region is still scarcely represented in most of the clinical databases worldwide or in the literature [98,99].

In our hands, the diagnostic yield of CMA as a second- or third-tier test for a cohort of patients with MCA from the Argentinian public health system was 21%. Our results were similar to reports from other populations in which microdeletions and microduplications have been described [6,7,13,14]. Nevertheless, the diagnostic yield of CMA depends on many factors, including the resolution of the platform used, patient selection criteria, sample size, previous testing performed, and the referring indication for testing.

None of the clinically relevant CNVs found in our cohort are strictly novel and most of the patients’ phenotypes were in accordance with known syndromes or were similar to previous reports in the literature. Nevertheless, and as we will discuss later, the fact that some of these CNVs presented different breakpoints may contribute to a better definition of the critical region involved in the diseases and/or to a better understanding of phenotypic variability among patients.

On the other hand, the high-resolution yield of almost 67% observed in our NGS analysis is mainly due to the implementation of the so-called phenotype-first approach in which the selection of patients for NGS analysis was based on the precise characterization of their phenotypes. An exhaustive phenotype-driven gene list of interest, developed in-house, also allowed the finding of clinically relevant genetic variants for most of the patients analyzed. As a promising result of our work, we found five novel pathogenic and likely pathogenic genetic variants described for the first time worldwide.

Some of the findings observed after CMA and NGS analysis deserve to be highlighted. As a consequence of a de novo unbalanced rearrangement, a newborn presented two pathogenic CNVs: a deletion in 1p36 and a duplication in 7q35. He presented some of the clinical characteristics already described in the 1p36 microdeletion syndrome, such as Ebstein’s anomaly [53,54,100] and hydrocephalus, which was previously described in patients with duplications in 7q [51,52] most probably due to an increased dose of *SHH* [101]. In addition, he presented VSD and intrauterine growth retardation, reported for both imbalances [53,54,102]. The child died in the neonatal period; the outcome could be a consequence of the synergy of the dose changes resulting from both imbalances.

We also found a newborn with a deletion in 8q21.11q21.3 that as well as presenting a phenotype compatible with 8q21.11 deletion syndrome (OMIM 614230) [40], also had hypoplastic left heart syndrome (HLHS), which has not been reported in patients with this syndrome. Of note is the fact that this patient also presented a balanced translocation between chromosomes 1 and 2 (Table 1). Although we were unable to detect imbalances involving this region, we cannot rule out the existence of any loss/gain of DNA material beyond the resolution range of the microarray platform used or in regions not included in this platform. Alternatively, the involvement of a positional influence of the translocated region in the development of the phenotype could also be considered [103]. Searching for pathogenic variants in genes related to HLHS could also be a suitable approach to perform in the near future to elucidate if additional genetic factors are involved in the clinical characteristics of this patient.

Omphalocele is one of the major ventral body wall defects. Chromosomal abnormalities have been reported in 10–12% of neonates and 30% of fetuses with omphalocele, respectively. We found a patient presenting omphalocele and a microdeletion in 2q14.2q14.3 encompassing the *GLI2* locus, a gene of the hedgehog signaling pathway. The correlation between omphalocele formation and this signaling pathway is controversial. In humans, *GLI2* pathogenic variants were associated with holoprosencephaly, oral cleft, polydactyly, and Culler-Jones Syndrome (OMIM 615849). Our patient had neither polydactyly nor oral cleft, and unfortunately, neuroimaging was not available to rule out features of the holoprosencephaly spectrum. Nevertheless, it should be considered that a familial *GLI2* deletion (2q14.2) has been described as not associated with the holoprosencephaly syndrome phenotype [44]. Moreover, mouse mutants of Sonic hedgehog (*Shh*), GLI-Kruppel family member 3 (*Gli3*) and Aristaless-like homeobox 4 (*Alx4*), members of the hedgehog signaling pathway, were involved in ventral body wall malformation especially in pups with omphalocele phenotypes [104]. Although *Gli2* was not particularly analyzed in that trial, it is known that in mammals the zinc finger containing transcription factors, *Gli1*, *Gli2*, and *Gli3* regulates the transcription of *Shh* responsive target genes [105,106]. Another report in murine models described an association between *Gli3*^−/−^ mutants and omphalocele, whereas left-sided congenital diaphragmatic hernia was observed in *Gli3*, *Gli2* and in a *Gli2/Gli3* double mutant [107]. Taking all these results together, we propose that the absence of *GLI2* may be related to the omphalocele exhibited by the patient.

A duplication of Xp22.33 that overlaps the regulatory region of the *SHOX* gene (short-stature homeobox-containing gene—OMIM 312865) was found in a patient with a transverse and terminal reduction defect in the left upper limb and complete abduction of the fingers of the hand. This region is located in the pseudoautosomal region of the X chromosome and is known to be responsible for short stature in Turner Syndrome. Duplications in this region have also been reported in Leri Weill Dyschondrosteosis (LWD OMIM 127300) and in idiopathic short stature (OMIM 300582) [45]. LWD is a skeletal dysplasia characterized by mesomelic disproportionate short stature and Madelung deformity of the wrist. The phenotype exerted by the patient, however, does not resemble LWS. Nevertheless, a report from Monzani et al., 2019 [108] describes a girl having a duplication on Xp22 born with terminal reduction of the right lower limb. The reduction comprised the absence of the lower leg and foot; she also had a supernumerary digit on the left foot. The duplication of Xp22 involved the two enhancer (upstream and downstream) regulatory regions of the *SHOX* gene. Additionally, she presented a duplication on 15q25.2. The authors suggested that short stature and the skeletal anomalies were attributable to the copy number variants in the regulatory *SHOX* region, along with the comorbidity of the growth hormone deficiency. They also suggested that the duplication on 15q25.2 might have contributed to the other clinical features shown in the girl, such as urogenital malformations. Our case and Monzani’s case agreed on a congenital terminal limb reduction associated with a duplication of regulatory regions of the *SHOX* gene, although they differ in whether the affected limb was upper or lower. Both cases could indicate that changes in the regulation of the *SHOX* expression could affect the normal development of the limbs, mainly in the proximal–distal axis.

We found a proband with a deletion in 16p12.2 presenting semilobar holoprosencephaly. Although the most common clinical findings in patients with this deletion are developmental delay, mild to profound cognitive impairment, growth impairment, cardiac malformations, epilepsy, and psychiatric and/or behavioral problems, it was suggested that the 16p12.2 recurrent deletion is characterized by variable phenotype that may not constitute a recognizable syndrome [50]. It was also suggested that the 16p12.2 recurrent deletion may represent an independent risk factor for severe neurodevelopmental phenotypes in association with other large pathogenic CNVs [50]. We did not find another clinically relevant CNV in the patient sample, but previous reports also described enrichment of rare likely pathogenic variants affecting functionally intolerant genes (“other hits”) [50]. Even though we cannot rule out the presence of a putative likely pathogenic variant, we recorded that the mother of the proband presented controlled gestational diabetes throughout her pregnancy which could account as well for the other hit proposed.

Patients carrying rare imbalances in 3p21.31 have been previously described; however, no recurrent breakpoints have been reported [62,63,64,65,109,110]. The presence of cortical blindness, central nervous system abnormalities, cleft lip, and intellectual disability, were summarized as the main characteristics of patients with deletions affecting this region [56]. The proband from our cohort with a de novo deletion in 3p21.31 presented cleft palate, atrial septal defect, VSD, growth and developmental delay, and short stature, among other clinical characteristics. These findings add to the current concept that imbalances within this genomic region are variable in size as well as the observed phenotypes.

In most infants, the etiology behind hydranencephaly (a congenital post-neurulation event) is usually unknown, although different mechanisms have been postulated for the disorder, such as vascular anomalies, neuroblast migration, or secondary infections [111]. In the present work, we described a female infant with hydranencephaly and a severe macrocephaly presenting a likely pathogenic deletion in 17q25.3 encompassing nine genes. One of the genes involved is *TBCD* (Tubulin folding cofactor D), which is one of the five tubulin-specific chaperones playing a pivotal role in microtubule assembly [112]. Even though *TBCD* was suggested to be related to early-onset progressive encephalopathy with brain atrophy and thin corpus callosum [49], a missense variant in *TUBA1A*, the gene encoding the α1a-tubulin, has been previously reported in a patient who had an extremely thin cerebral parenchyma resembling hydranencephaly. Functional assays of the affected protein showed that the mutated microtubules are less stable than the normal ones [113]. In line with previous reports related to tubulinopathy [114], and considering that genes encoding cytoskeletal proteins are important in the developing brain, we propose that the loss of *TBCD* may be related to our patient’s phenotype by altering the normal functioning of microtubules in neuroblast cells. The altered function could interfere with normal proliferation and migration processes at the time of prenatal development with the consequent cortical alteration leading to a hydranencephaly or hydranencephaly-like phenotype.

Among the novel variants found after NGS analysis, a novel variant p.H270Y in the *SHH* gene was identified in a patient presenting cyclopia with proboscis and cryptorchid. *SHH* is one of the most important morphogens in animals. It is involved in the pattern formation of limbs and the ventral midline structure of the central nervous system [115] and is one of the well-known holoprosencephaly responsible genes [42]. It is expressed as a precursor protein that undergoes autoproteolysis; a process that is essential for full biological activity [116]. The p.H270Y is a missense change affecting a key and conserved residue suggested to be involved in this process. Indeed, it was demonstrated that by changing this histidine residue in the Drosophila orthologous gene *Hh*, the autoproteolysis is blocked, impairing its function [67].

In addition, we find a 5 bp deletion in the *MYH11* gene in a compound heterozygous state with a deletion of 0.6 Mb in 16p13.11 containing this gene in two probands affected with MMIHS. Indeed, this region has been previously described in patients with MMIHS in a compound heterozygous state with a loss of function variant in *MYH11* on the homologous allele [46,47,117]. This 5 bp deletion involved the canonical acceptor splicing site and could alternatively generate exon 26 exclusion or the putative usage of another cryptic acceptor splicing site (in exon 26 or even in the intron 26) leading either to a frameshift with the occurrence of a premature stop codon or to an in-frame indel. Whereas homozygous or compound heterozygous loss-of-function variants in *MYH11* cause MMIHS, heterozygous pathogenic variants account for 2% of Thoracic Aortic Aneurysm and Dissection (TAAD), and are also often associated with patent ductus arteriosus [47,68,117,118,119,120]. Considering the heterozygous condition of the parents, a close follow-up will be conducted.

In one of the patients recruited with MCA and familial intestinal polyposis, we found a 0.02 Mb deletion in the *APC* gene to be the main genetic cause for adenomatous familial polyposis [57,58], and a pathogenic variant p.(Gln2361Ter) in the *EP300* gene. Variants in *EP300*, mostly in the HAT domain, are associated with Rubinstein Taybi 2 syndrome (RTS2, OMIM 613684). On the other hand, variants located outside this protein domain may lead to a broad spectrum of phenotypes [74,75], and particularly, patients with frameshift variants reported in the carboxy-terminal of the protein exhibited a mild phenotype [74,121]. The variant found in our patient is located in exon 31 in the carboxy-terminal of the protein outside the HAT domain, is a nonsense variant, and is predicted to generate a truncated protein. Nevertheless, his phenotype most likely resembled the clinical characteristics often seen in RTS2, such as postnatal growth retardation, microcephaly, intellectual disability, cardiovascular anomalies, urinary tract anomalies, and cervical vertebral abnormalities [74,76]. The p.(Gln2361Ter) was also found in the father who only had intestinal polyposis. Despite the fact that most of the EP300 variants reported are de novo [121] may argue against the pathogenicity of the p.(Gln2361Ter) variant, two patients have been described with inherited genetic variants, one from a milder symptomatic mother [121] and the second from an asymptomatic father [74]. Altogether, these observations may indicate a possible incomplete penetrance for genetic variants in *EP300*.

*MYH7* is a gene widely reported in the literature as a cause of familial hypertrophic/dilated cardiomyopathy (MIM 192600) and left ventricular noncompaction (LVNC) cardiomyopathy (MIM 613426), with more than 200 clinically relevant variants described so far [84] (www.varsome.com, last entry 20 March 2022). The novel p.(Asn224Ile) missense variant found in our patient is located in the myosin motor domain of the protein and predicted as deleterious. The myosin motor domain encompasses 693 amino acids out of 1935 in the protein, and most of the pathogenic variants described are located in this domain.

Finally, an interesting finding after NGS analysis is the presence of the likely pathogenic variant p.(Arg726Cys) in the *MYBPC3* gene in two unrelated patients, one exhibited noncompaction cardiomyopathy and the other Tetralogy of Fallot (ToF) as part of a phenotype with MCA. *MYBPC3* is a well-known gene for cardiomyopathy, and the p.(Arg726Cys), as other genetic variants, has been previously described in patients with this condition [122,123,124,125,126,127]. To a lesser extent, deleterious genetic variants in this gene were also previously reported in two patients with ToF, together with other VUS in *SOS1* and in *ARVCF*, respectively [82,83]. In line with these observations, our results may add to the concept that cardiomyopathy genes may contribute to a genetic basis of ToF [83]. In addition, the proband had partial agenesis of the corpus callosum and renal agenesis. Whereas no clinically relevant CNV or another genetic variant were found, we cannot rule out the involvement of variants in other genes not included in our phenotype-driven gene list of interest, and/or the influence of environmental factors during pregnancy that may influence the development of the CA in the child.

## 5. Conclusions

Using an algorithm that combines molecular techniques with clinical and genetic evaluation, we were able to determine the genetic contribution in 27.5% of the patients: 30% of the analyzed patients with MCA and 23.7% of the patients with isolated CHD. After conventional cytogenetic studies, chromosomal anomalies were found in 12.6% of patients with MCA. In addition, imbalances in the 22q11 were found in 20.5% of the patients studied, including patients presenting isolated conotruncal CHD. The diagnostic yield of CMA, as a second- or third-tier test, was 21%. This technique was also useful for failed karyotypes, allowing us to define the genetic etiology in patients that would have otherwise remained undiagnosed. Finally, and based on a phenotype-first approach, 67% of the patients analyzed by NGS presented a clinically relevant genetic variant, five of them described for the first time worldwide. These findings, together with the description of the clinically relevant CNVs found in affected individuals from our cohort, add to the knowledge of the putative molecular mechanism involved in the development of these diseases and to the characterization of the genetic basis in affected individuals with CA from our population. Our study also evidences the need for interdisciplinary work between the congenital anomalies surveillance system, public health effectors and high-complexity laboratories, to extend genetic diagnosis and counseling accessibility in our country. Also, it would be important to articulate these initiatives in the formulation of health policies that may contribute to improving care and prevention of congenital anomalies.

## Figures and Tables

**Table 1:** Cytogenetic studies of patients with MCA.

Karyotype	N	N (%)
46,XX	51	89 (86.4)
46, XY	38	
47,XY,+13	1	13 (12.6)
47,XY,+18 or 47,XX,+18	6	
47,XXX [28]/47,XX,+14 [12] ^1,2^	1	
46,XY,t(1;2)(q25;q21) ^1^	1	
46,XX,t(11;17)(p10;p10)	1	
46,XX,del(15)(q11.2q13) ^3^	1	
47,XY,+idic(18)(p10) ^3^	1	
46,XY,trp(8)(p21.2p21.1) ^1^	1	
Total	103	103 (100)

^1^: These patients were also analyzed by array-CGH (refer to Table 3 for details). ^2^: Described in Massara et al., 2019 [31]; ^3^: Confirmed by FISH.

**Table 2:** MLPA analysis in patients with CCHD or suspected 22q11DS.

Imbalances	Conotruncal CHD		Suspected 22q11DS ^1^		Total
	With MCA	Isolated CHD	With MCA	Isolated CHD	
None	18	60	15	12	105
del(22)(q11) 3 Mb	5	13	-	3	21
del(22)(q11) 1.5 Mb	1	2	-	-	3
dup(22)(q11) 1.5 Mb	1	1	-	-	2
rsa22q11.2 (TBX1-7x1)		1	-	-	1
Total	25	77	15	15	132

CHD: Congenital Heart Diseases; MCA: Multiple Congenital anomalies; del: deletion; dup: duplication; 22q11DS: 22q11 deletion syndrome. ^1^: without conotruncal CHD. Partial results of this MLPA analysis have been published in Delea et al., 2018 [28].

**Table 4:** Clinically relevant genetic variants found by NGS analysis.

Patient ID	Phenotype	Gene	Sequencing Technology	Genetic Variant	Protein Change	Classification	Supporting Evidence
57	MCA	*SHH*	WES	**(NM_000193.4):c.808C>T**	p.His270Tyr	Likely Pathogenic	[67]
100	MCA	*MYH11*	WES	**(NM_001040114.1):c.3143-2_3145delAGTGC**	p.?	Pathogenic	[47,68]
114	MCA	*PTPN11*	TSC	(NM_002834.5):c.1381G>A	p.(Ala461Thr)	Pathogenic	[69,70]
123	MCA	*FOXL2*	WES	(NM_023067.4):c.644A>G	p.(Tyr215Cys)	Pathogenic	[71,72]
129	MCA	*PTPN11*	WES	(NM_002834.5):c.922A>G	p.Asn308Asp	Pathogenic	[73]
149	MCA	*EP300*	WES	**(NM_001429.4):c.7081C>T**	p.(Gln2361Ter)	Pathogenic	[74,75,76]
175	MCA	*PTPN11*	TSC	(NM_002834.35)c.181G>A	p.(Asp61Asn)	Pathogenic	[77,78,79,80]
188	MCA	*KAT6B*	WES	**(NM_012330.4):c.4572_4573dupTA**	p.(Thr1525IlefsTer25)	Pathogenic	[81]
232 ^1^	MCA	*MYBPC3*	WES	(NM_000256.3):c.2176C>T	p.(Arg726Cys)	Likely Pathogenic	[82,83]
333	iCHD	*MYH7*	TSC	**(NM_000257.4):c.671A>T**	p.(Asn224Ile)	Likely Pathogenic	[84]
335	iCHD	*RAF1*	TSC	(NM_002880.3):c.770C>T	p.(Ser257Leu)	Pathogenic	[85]
351 ^1^	iCHD	*MYBPC3*	TSC	(NM_000256.3):c.2176C>T	p.(Arg726Cys)	Likely Pathogenic	[86]

Novel variants are bolded. MCA: Multiple Congenital anomalies; iCHD: isolated Congenital Heart Disease; ^1^: Unrelated patients. WES: Whole exome sequencing followed by an in-silico selection of candidate genes for variant analysis. TSC: TruSight^®^ Cardio Sequencing kit.

## Data Availability

All clinically relevant results are publicly available in this paper. Clinically relevant CNVs and genetic variants were uploaded to the ClinVar database (accession # SCV002074131, SCV002098068–SCV002098099, SCV002098932).

## Supplementary Materials

The following are available online at https://www.mdpi.com/article/10.3390/genes13071172/s1: Figure S1: Ideogram of the clinically relevant copy number variations (CNVs) found by array-chromosomal genomic hybridization (array-CGH); Figure S2: Representative electropherograms of Sanger sequencing of patients in whom clinically relevant genetic variants have been found; Table S1: Phenotypes of patients with clinically relevant CNVs; Table S2: Imbalances classified as VUS in patients with MCA; Table S3: Phenotypes of patients with clinically relevant genetic variants.
