# Supplementary material for: Genetic Analysis Algorithm for the Study of Patients with Multiple Congenital Anomalies and Isolated Congenital Heart Disease
Source: Genes (Basel). 2022 Jun 29;13(7):1172. doi: 10.3390/genes13071172 (PMC9317700; doi:10.3390/genes13071172)
Supplement: Supplementary file 1 [file genes-13-01172-s001.zip › genes-1728382-supplementary.pdf]

SUPPLEMENTARY MATERIAL

Genetic analysis algorithm for the study of patients with Multiple Congenital Anomalies and isolated Congenital Heart Disease†

Marisol Delea[1], Lucia S. Massara[2], Lucia D. Espeche[1], María Paz Bidondo[1,3], Pablo Barbero[1], Jaen Oliveri[2&], Paloma Brun[2], Mónica Fabro[4], Micaela Galain[4], Cecilia S. Fernández[4], Melisa Taboas[1], Carlos D. Bruque[1&], Jorge E. Kolomenski[5], Agustín Izquierdo[6], Ariel Berenstein[7], Viviana Cosentino[8], Celeste Martinoli[9], Mariana Vilas[10], Mónica Rittler[10], Rodrigo Mendez[1], Lilian Furforo[10], Rosa Liascovich[1], Boris Groisman[1], Sandra Rozental[1], Liliana Dain[1,8*] and the PID ACM-CC group.

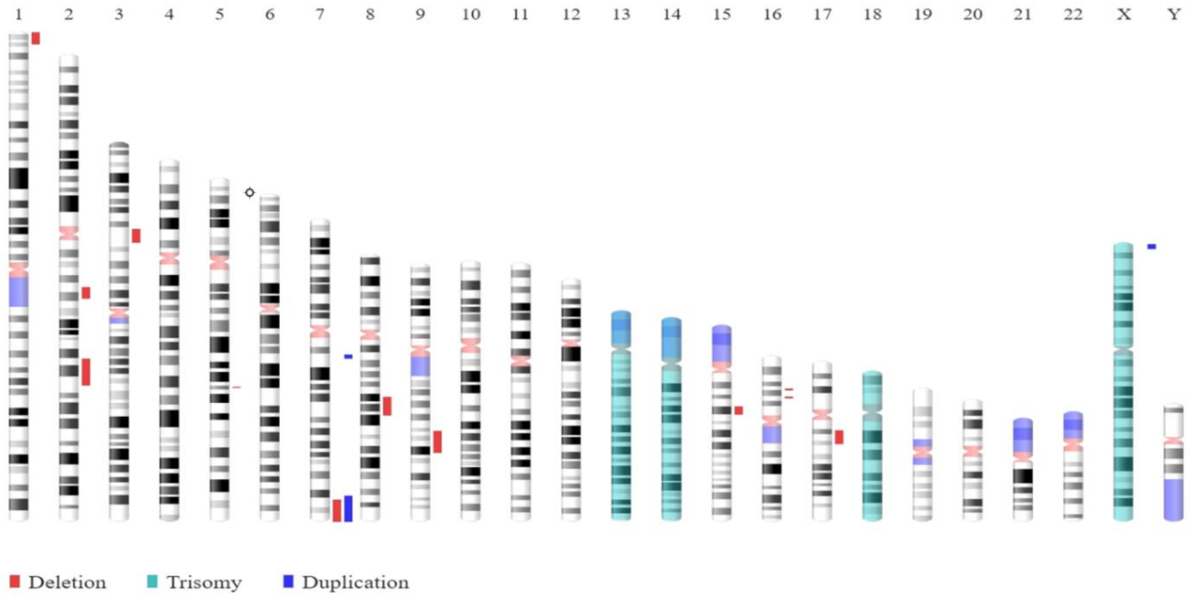

Figure S1: Ideogram of the clinically relevant copy number variations (CNVs) found by array-chromosomal genomic hybridization (array-CGH)

SHH

ID 57:c.808C>T

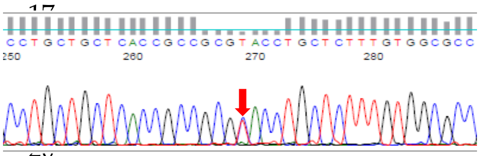

PTPN11

ID 114:c.1381G>A

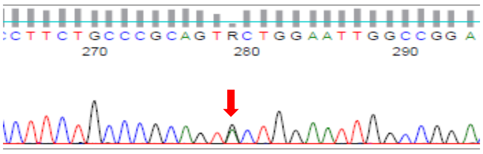

MYH11

ID 100: c.3143-2_3145delAGTGC; Hom

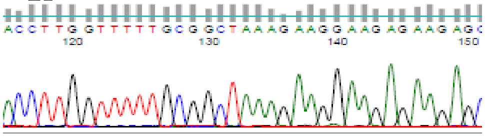

ID 100H1:c.3143-2_3145delAGTGC; Hom

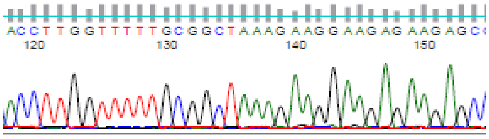

ID 100 Father

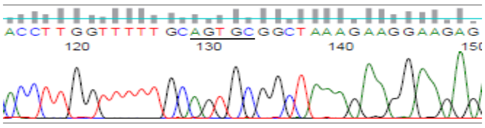

ID 100 Mother: c.3143-2_3145delAGTGC; Het

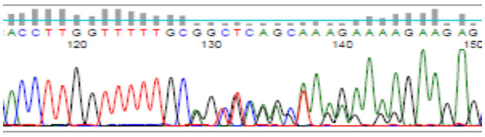

FOXL2

ID 123 c.644A>G

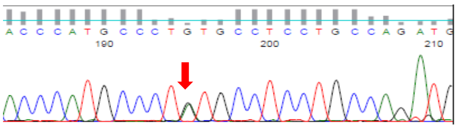

ID: 123 Father : c.644A>G

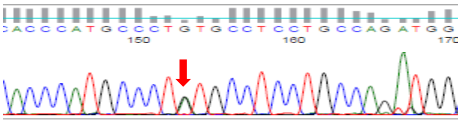

ID 123 Mother

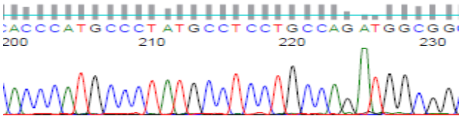

PTPN11

ID 129:c.922A>G T

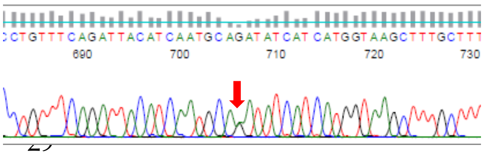

PTPN11

ID 175 c.181G>A

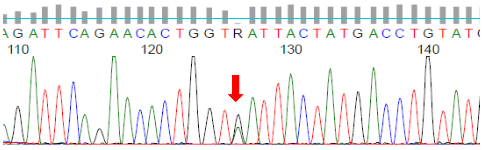

EP300

ID 149 (NM_001429.4):c.7081C>T

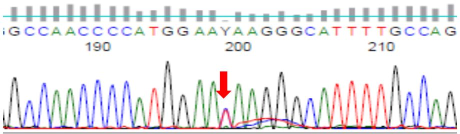

ID 149 Father (NM_001429.4):c.7081C>T

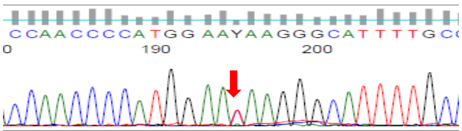

ID 149 Mother

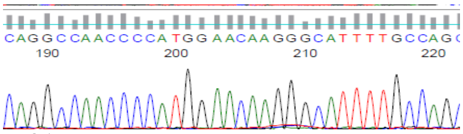

MYBPC3

ID 232 c.2176C>T

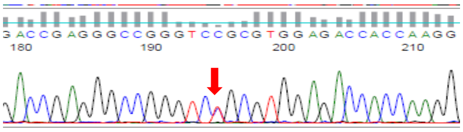

ID 232 Father

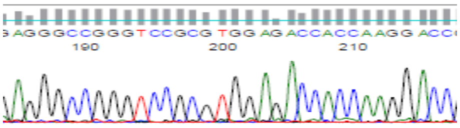

ID 232 Mother c.2176C>T

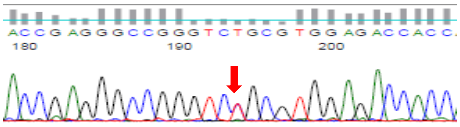

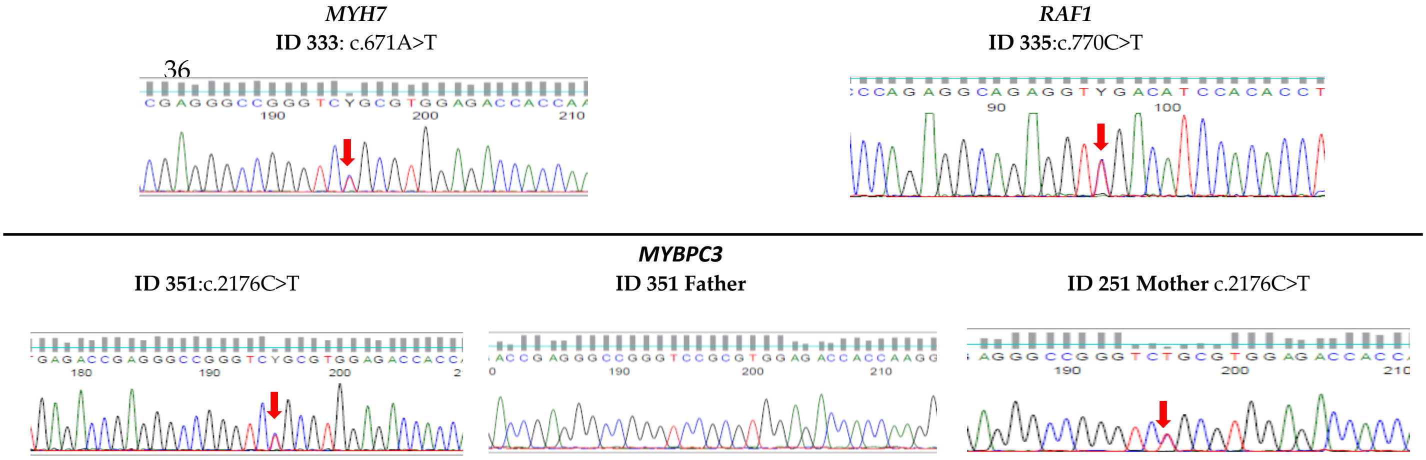

Figure S2: Representative electropherograms of Sanger sequencing of patients with clinically relevant genetic variants. Red arrows indicate the position of the variants. The horizontal line in ID 100 Father represents the 5 bp deletion found in the probands. Hom: homozygous; Het: Heterozygous. The electropherogram of the patient ID 188 with the **c**.4572_4573dupTA genetic variant in *KAT6B* gene is not included since it have been published previously (Mendez, R.; Delea, M.; Dain, L.; Rittler, M. A Novel Pathogenic Frameshift Variant of KAT6B Identified by Clinical Exome Sequencing in a Newborn with the Say–Barber–Biesecker–Young–Simpson Syndrome. *Clin. Dysmorphol.*2020, *29*, 42, doi:10.1097/MCD.0000000000000270).

Table S1: Phenotypes of patients with the clinically relevant CNVs

Patient ID	Genital Gender	Phenotype
2	F	Muscular VSD, ASD, IAA Type B, PDA, microphthalmia, short palpebral fissures, low set ears. Short neck, retrognathia, separate areolas, micrognathia
41	M	Bilateral talipes equinovarus, partial agenesis of the corpus callosum, PDA (gestational age 41 weeks , 3630g)
481	M	HLHS, low set ears, microphthalmia
65	M	Microcephaly, cyclopia, proboscis, semilobar holoprosencephaly with thalamic fusion, univentricular and with a single choroid plexus
68	M	Omphalocele, hypoplastic right colon, small intestine atresia (type IIIA), slight pyelocalyceal dilation, preterm newborn, low bith weight for gestational age, IUGR (type 1)
94	F	Intrauterine growth restriction, high anterior hair implantation, absence of eyebrows, narrow palpebral fissures, bilateral iris coloboma, high and wide nasal bridge, neonatal tooth, cleft hard and soft palate, Pierre Robin sequence, asymmetric grade II microtia, Breast hypertelorism, left hand with ulnar deviation, long fingers, finger pads, bilateral clinodactyly, anterior anus,ToF, pathological fundus with pigment dispersion
96	F	Skin tag on the right cheek, absent left external auditory canal, transverse and terminal reduction defect in the left upper limb and complete abduction of the fingers of the hand.
1002	F	Megacystis, microcolon, hypoperistalsis syndrome, ASD
106	F	Severe macrocephaly (head circumference of 44 cm, in +5 SD according to gestational age), large anterior and posterior fontanelle, forehead, intermittent horizontal nystagmus and ocular retroversion, unilateral preauricular pit without other facial dysmorphism. Scant frontal cortical tissue with the absence of the midline suprathalamic structures compatible with hydranencephaly. Anterior anal displacement and kidney pyelocaliceal dilation
127	M	Umbilical hernia, inguinal hernia, bilateral 5th finger clinodactyly, bilateral transverse palmar crease, 4th metacarpal hypoplasia, semilobar holoprosencephaly.
134	M	IUGR, Ebstein's anomaly, VSD, suture diastasis, wide fontanelles, dysplastic ears. Communicating hydrocephalus. Asymmetric lateral ventricles. Asymmetric ventricular walls. Left subependymal bilocular cyst.
147	M	Brachyplagiocephalia, broad forehead, narrow palpebral fissures, short nose, broad tip and broad nostrils, patterned philtrum, cleft hard and soft palate, lowered ears, short neck, PS, overlapping toes, transverse plantar groove. Dandy Walker vs cerebellar vermis hypoplasia, pyelocalyceal dilation. Repetitive horizontal movements of the head, slight maturational delay.

Patient ID	Genital Gender	Phenotype
149	M	Short stature, microcephaly, short palpebral fissures, bushy eyebrows, malar hypoplasia, long nose, prognathism, narrow palate, small mouth, multiple oral frenula, winged scapula, radioulnar synostosis, left brachial paresis (probably post surgical), reducible camptodactyly in both hands, general joint stiffness, bilateral inguinal hernia, VSD, PS, unilateral renal agenesis, intestinal polyposis, cervical vertebrae fusion, general decreasement of fat tissue, muscular hypertrophy, mental retardation (mild to moderate)
167	M	Complete bilateral cleft of the lip, sparse eyebrows, left eye retinal coloboma, small palpebral fissures, perimembranous VSD, CoA, kidneys with increased size and poor corticomedullary differentiation, probable polycystic kidney disease, bilateral cryptorchidism. Hypocalcaemia
187	F	Macrocephaly, hydrocephalus. microstomia, narrow palate, bilateral ear agenesis, bilateral agenesis of the ulna and radius, bilateral agenesis of the thumbs, rocking feet, open sacral myelomeningocele, hypoplasia of the left cavities, right renal agenesis, and agenesis of the bladder
233	M	Trigonocephaly, cryptorchidism and basal cell carcinoma
362	M	Cleft palate, dysmorphic facial features, ASD, VSD, clinodactyly of the 5th finger, growth delay, short stature, hypertelorism, upslanting palpebral fissure , flat nasal bridge , micrognathia, downturned corners of the mouth , developmental delay , gastroesophageal reflux
368	M	Agenesis of the corpus callosum, asymmetry of the cardiac chambers, dilatation of the right chambers, perimembranous VSD

M: Male; F: Female; VSD: Ventricular septal defect; ASD: Atrial septal defect; IAA: Interrupted Aortic Arch; PDA: Patent ductus arteriosus; PS. Pulmonary stenosis; HLHS: Hypoplastic left heart syndrome; CoA: Coarctation of the aorta; IURG: Intrauterine growth retardation; ToF: Tetralogy of Fallot.

Table S2: Imbalances classified as VUS in patients with MCA

Patient ID	Genital Gender	Phenotype	Karyotype	Imbalance	Size (Mb)
37	F	IURG, microretrognathia, low-set ears. palpebral fissures asymmetry, wide neck. hypertelorism, short and upturned nose. Distal phalanges and very short nails. Mild to moderate bilateral ventriculomegaly. Left eye microphthalmia, microcornea. Coloboma of the retina, choroid and optic nerve.	46,XX	arr[GRCh37] 4q13.3(73694233_73984429)x3	0.29
104[1]	M	Transverse and terminal reduction defect in the left upper limb (presents 3/4 parts of the radius and ulna), absence of phalanges, metacarpals and carpus. VSD.	Failed	arr[GRCh37] 5q15(94478206_94864722)x1	0.39
203	M	Low dorsal myelomeningocele (5 x 5 cm), hydrocephalus, rib fusion, left rib agenesis,PDA.	46,XY	arr[GRCh37] 3p26.3(1125700_1197682)x1	0.07
211	M	Unilateral cleft lip alveolus and palate, hypertelorism, low-set ears, Tricuspid atresia, IAA, VSD	46,XY	arr[GRCh37] 20p13(489033_898472)x3	0.41
329	F	Retrognathia, wide nose, low hairline, asymmetric bite, cleft palate, short and dysfunctional soft palate, PS, thoracic vertebral segmentation defect, neurodevelopmental delay.	46,XX	arr[GRCh37] 19q13.42q13.43(55434660_56463734)x1	1,03
349	M	IURG, facial dysmorphism, subaortic VSD, hypospadias, inguinal hernia.	Failed	arr[GRCh37] 9p24.3(204193_349093)x3,	0,14

[1]: Analyzed with ISCA 4x180. M: Male; F: Female; IURG: Intrauterine growth retardation; VSD: Ventricular septal defect; PDA: Patent ductus arteriosus IAA: Interrupted Aortic Arch; PS: Pulmonary stenosis

Table S3 : Phenotypes of patients with clinically relevant genetic variants

Patient ID	Genital Gender	Phenotype
57	M	Cyclopia with proboscide, cryptorchidie
100[1]	F	Megacystis, microcolon, hypoperistalsis syndrome, ASD
100H1[1]	F	Megacystis, microcolon, hypoperistalsis syndrome, ASD
114	M	Asymmetric hypertrophic septal cardiomyopathy, subaortic stenosis, hypertelorism, right cryptorchidism, epicanthus, low pinna implantation
123	F	IUGR, BPES, cleft palate, retrognathia, small ears, high anterior hairline implantation, hands with long fingers, cutaneous umbilicus, generalized decrease in subcutaneous cell tissue, asymmetric sacral folds, slightly anterior anus.
129	M	PS, ASD, suspected noonan syndrome. Relative macrocephaly, wide forehead, descending palpebral fissures, slightly spread ears, fine nose, bulbous tip, short neck without pterygium, pectus excavatum (sternotomy), unilateral cryptorchidism, linear hyperpigmentation on the inner side of the right arm and forearm following the line of blaschko, scoliosis
149	M	See Table S1
175	M	PFO, VSD, PS, excess skin on the nape, transverse fold in the left bridge, shield chest, pectus excavatum, bilateral cryptorchidism, small rough pigmented scrotum. Suspected Noonan syndrome
188	M	Expressionless face, short, upslanting palpebral fissures, ptosis, epicanthus inversus, high nasal bridge and rounded tip, small low-set ears, small mouth with downturned corners and mostly open cleft palate ,severe microretrognathia, optic nerve hypoplasia, broad hands with thumbs and halluces remarkably long, VSD, hypoplastic genitalia, bilateral cryptorchidism, seizures, hypotonic, presented feeding and respiratory difficulties requiring tracheostomy
232[2]	F	Partial agenesis of corpus callosum, ToF, renal agenesis
333	F	Noncompaction cardiomyopathy
335	F	Prominent forehead with flat capillary malformation, sparse hair, descending palpebral fissures, low nasal bridge, slight retrognathia, lowered and rotated ears. Hypertrophic cardiomyopathy. Resembling Costello syndrome phenotype
351[2]	F	Noncompaction cardiomyopathy

[1]: Siblings.[2]: Unrelated patients M: Male; F: Female; ASD: Atrial septal defect; BPES: Blepharophimosis, Ptosis, Epicanthus Inversus syndrome. PS: Pulmonary stenosis; VSD: Ventricular septal defect; PFO: Patent foramen ovale; IURG: Intrauterine growth retardation; ToF. Tetralogy of Fallot
